# The accuracy of forecasted hospital admission for respiratory tract infections in children aged 0–5 years for 2017/2023

**DOI:** 10.3389/fped.2024.1419595

**Published:** 2025-01-06

**Authors:** Fredrik Methi, Karin Magnusson

**Affiliations:** ^1^ Cluster for Health Services Research, Norwegian Institute of Public Health, Oslo, Norway; ^2^ Clinical Epidemiology Unit, Orthopaedics, Department of Clinical Sciences Lund, Faculty of Medicine, Lund University, Lund, Sweden

**Keywords:** respiratory tract infection, COVID-19, respiratory syncytial virus, hospital admission, health care use, immunity debt

## Abstract

**Aim:**

Healthcare services are in need of tools that can help to ensure a sufficient capacity in periods with high prevalence of respiratory tract infections (RTIs). During the COVID-19 pandemic, we forecasted the number of hospital admissions for RTIs among children aged 0–5 years. Now, in 2024, we aim to examine the accuracy and usefulness of our forecast models.

**Methods:**

We conducted a retrospective analysis using data from 753,070 children aged 0–5 years, plotting the observed monthly number of RTI admissions, including influenza coded RTI, respiratory syncytial virus (RSV) coded RTI, COVID-19 coded RTI, and other upper and lower RTI, from January 1st, 2017, until May 31st, 2023. We determined the accuracy of four different forecast models, all based on monthly hospital admissions and different assumptions regarding the pattern of virus transmission, computed with ordinary least squares regression adjusting for seasonal trends. We compared the observed vs. forecasted numbers of RTIs between October 31st, 2021, and May 31st, 2023, using metrics such as mean absolute error (MAE), mean absolute percentage error (MAPE) and dynamic time warping (DTW).

**Results:**

In our most accurate prediction, we assumed that the proportion of children who remained uninfected and non-hospitalized during the lockdown would be prone to hospitalization in the subsequent season, resulting in increased numbers when lockdown measures were eased. In this prediction, the difference between observed and forecasted numbers at the peak of hospitalizations requiring vs. not requiring respiratory support in November 2021 to January 2022 was 26 (394 vs. 420) vs. 48 (1810 vs. 1762).

**Conclusion:**

In scenarios similar to the COVID-19 pandemic, when the transmission of respiratory viruses is suppressed for an extended period, a simple regression model, assuming that non-hospitalized children would be hospitalized the following season, most accurately forecasted hospital admission numbers. These simple forecasts may be useful for capacity planning activities in hospitals.

## 1 Introduction

The COVID-19 pandemic impacted countries worldwide in diverse ways (1, 2). While Norway experienced comparatively low hospitalization and mortality rates (3), rigorous measures were implemented (4). These interventions were aimed at reducing mortality, but also to safeguard the capacity of the healthcare system, preventing it from being overloaded by a potential surge in COVID-19 cases (4).

Between November 2021 and January 2022, there were signs that hospital capacity for children with respiratory tract infections (RTIs) such as upper and lower RTIs, influenza coded RTI, respiratory syncytial virus (RSV) coded RTI, and COVID-19 coded RTI in Norway was threatened. To aid health authorities and hospitals in their planning of healthcare activities, we projected the expected number of hospital admissions among children aged 0–5 years in Norway due to these RTIs (5). Specifically, we analyzed the data from 2017 to the beginning of the 2021/2022 RTI season and forecasted hospital admissions for various types of RTIs, as well as the number of children who required respiratory support, up until May 2023.

There is a need to retrospectively investigate the usability and correctness of the forecast models, as they may be used for hospital planning in future scenarios when there is a similar uncertainty as ruled during the COVID-19 pandemic. Earlier forecast models for RTI admissions, published prior to the COVID-19 pandemic, were mainly based on meteorological parameters (6–8), which may not be feasible in or after a pandemic that is being caused by a new virus and independent of seasonal variation. Forecast models that were developed and investigated during the COVID-19 pandemic were primarily based on mobility data or aggregated data from mobile networks (9, 10). These data are typically challenging to obtain or use for hospital planners.

There is a need for simple models that are easily available in hospital planners' daily practice, for example models that are based on cause-specific hospital admissions. Existing forecast models based on easily accessible data, for example in-hospital-gathered data, are well worked through statistically and provide promising and accurate tools in the short-term setting (11, 12). However, these models have limitations; they are often not applicable to periods beyond a 7-, 14-, or 21-day periods, which is typically too short for action (13); they are often limited to COVID-19 admissions or other specific pathogens and not easily applicable to other RTIs; and they are to a limited extent accessible and understandable to a layman audience (11, 12).

We found no forecast models that include several RTIs that typically affect young children. Young children, including infants and toddlers up to age 5 are in a particularly vulnerable situation in and after a pandemic where strict disease control measures are implemented, because they have not yet fully developed their immunity systems (14). For example, due to the lack of exposure to typically circulating viruses, young children may be hypothesized to experience a greater lack of immunity, often referred to as “immunity debt” or “immunity gap” (14–17), when non-pharmaceutical interventions (NPIs) such as school closures or lockdowns are eased, compared with older children and adults. This effect may be heightened for infants, who may lack antibodies passed on by their mothers through breastfeeding or the placenta (18–20). With the knowledge that RTIs caused by other pathogens than SARS-CoV-2 will result in the most severe long-term disease in young children (21), forecast models for young children should include all common reasons for hospital admission (upper and lower RTI, influenza, RSV, and COVID-19).

In the present study, conducted between January 1st and April 18th, 2024, we aim to retrospectively investigate the observed numbers of hospitalized children with RTIs, and thus evaluate the accuracy of our predictions and usefulness of our forecast methods for future pandemics.

## 2 Materials and methods

### 2.1 Design and data sources

We used a similar design and data sources as published for the forecast study (5), namely the nationwide Norwegian emergency preparedness register, Beredt-C19 (22). This register includes daily individual-level data from several registers, including the Norwegian Patient Register (NPR) (all electronic patient records from all hospitals in Norway) and the National Population Register (birth date and sex). An institutional board review was conducted, and the Ethics Committee of South-East Norway confirmed (June 4th, 2020, #153204) that external ethical board review was not required.

### 2.2 Population

Our population consisted of an open cohort with all children aged 0–5 years during the given month who were registered as Norwegian residents. Children who were born (stillbirths not included) or who immigrated were included in both the numerator and denominator from the first full month following the date of birth or immigration (and similarly excluded in the month of their death or emigration, which was extremely rare). In line with our previous study (5), children were included the following calendar month after birth and excluded the calendar month they turned 6 years old. In total we included 753,070 unique children.

### 2.3 Outcome: RTI and COVID-19

We considered all inpatient hospital visits regardless of length of stay and urgency. If hospital contacts occurred within less than 48 h of each other, we considered them as the same admission. Similarly, outpatient contacts occurring less than 48 h before or after an inpatient admission were included as the same admission. In cases where multiple diagnostic codes were registered, we selected the code with the highest specificity (i.e., with known pathogen if available). Based on an article by Reeves et al. from 2020 (23), we focused on 66 International Classification of Diseases (ICD-10) codes related to both viral and unspecified RTIs (5). We categorized these codes into five mutually exclusive categories: Other upper RTI (known pathogens and unspecified), other lower RTI (known pathogens and unspecified), influenza coded RTI, RSV coded RTI, and COVID-19 coded RTI, as presented in Table 1. We also analyzed the healthcare needs of these categories of diagnoses by examining whether they required respiratory support.

**Table 1:** ICD-10 codes and RTI categories.

Categories	ICD-10 codes
Other upper RTI	J00 **J02.0 J02.8** J02.9 **J03.0 J03.8** J03.9 J04.0 J04.1 J04.2 J05.0 J05.1 J06.0 J06.8 J06.9
Other lower RTI	**J12.0 J12.2 J12.3 J12.8** J12.9 **J13 J14 J15.0 J15.1** **J15.2 J15.3 J15.4 J15.5 J15.6 J15.7 J15.8** J15.9 **J16.0** **J16.8 17.0 J17.1 J17.2 J17.3 J17.8** J18.0 J18.1 J18.2 J18.8 J18.9 J22 **J20.0 J20.1 J20.2 J20.3 J20.4 J20.6** **J20.7 J20.8** J20.9
Influenza coded RTI	**J09 J10.0 J10.1 J10.8** J11.0 J11.1 J11.8
RSV coded RTI	**J12.1 J20.5 J21.0**
COVID-19 coded RTI	U07.1 U07.2
Respiratory support	GXAV01 GXAV10 GXAV30

Note: Diseases with known pathogen in bold. Codes for respiratory support: Ventilation (GXAV01); Continuous Positive Airway Pressure (CPAP) (GXAV10); High flow oxygen therapy (GXAV30).

## 3 Statistical analysis

First, we plotted the observed monthly number of all RTI admissions, with and without respiratory support and for each of the included types of RTI, from January 1st, 2017, to May 31st, 2023. Second, we compared the numbers of RTI hospitalizations with and without respiratory support, against each of the four forecasted scenarios.

### 3.1 Scenarios

In scenario (1) “Business as usual”, we assumed that the pandemic would not have a lasting impact on the number of RTI hospitalizations beyond the 2020–2021 RTI season. Therefore, we assumed that the trend observed from 2017 to 2019 would persist from the fall of 2021. Our estimates were derived using a simple ordinary least squares (OLS) regression incorporating a monthly time trend, expressed as $y_{t}=\;\beta_{0}+\;\beta_{1}\cdot k_{t}+\;\beta_{2}\cdot m_{1}+\beta_{3}\cdot m_{2}+.\;.\;.\;+\;\beta_{12}\cdot m_{11}\;+$ *ɛ*_*t*_, where *t* signifies the month; $m_{1}$ equals 1 for January and 0 for other months; $m_{2}$ equals 1 for February and 0 otherwise, and so on, with December serving as the reference month. The model also incorporated a potential time trend in hospital admissions over time, $k_{t}$, where $k_{1}=1$ in January 2017, $k_{2}=1\;$ in February 2017, and so forth. To establish the trend for “Business as usual”, we utilized data from January 2017 (*t* = 1) to December 2019 (*t* = 36) and forecasted the data from August 2021 onward.

Scenario (2) “Continuous lockdown” was based on the assumption that the pandemic and NPIs had a permanent impact on the number of RTI hospitalizations, nearly eradicating the diseases. We employed the same formula as in scenario 1 for this calculation. However, unlike the previous scenario, we did not establish the trend using observations from 2017 to 2019. Instead, we calculated the trend using data from July 2020 (*t* = 43) to June 2021 (*t* = 54), when lockdown restrictions were in place.

In scenario (3) “Children's immunity debt”, we assumed that RTI hospitalizations were only temporarily affected during the pandemic lockdown measures. We further assumed that the numbers of non-hospitalized children during the lockdown would contribute to an “immunity debt” (5, 14–17), and that the same number of non-hospitalized children during the lockdown would come in addition to the normal number of hospitalized children the next season. To calculate this, we utilized a similar regression as in scenario 1, but incorporated the number of “spared” hospitalizations during the 2020–2021 season. These hospital admissions were then distributed across the months. Technically, we computed the difference between the number of estimated hospital admissions from scenario 1 and the number of observed hospital admissions from January 2020 (*t* = 37) through July 2021 (*t* = 55). We then divided the sum by each calendar month's average share of hospital admissions per season, and added them on top of the trend from scenario 1.

In the fourth, and final, scenario (4) “Maternal immunity debt”, we applied similar assumptions as in scenario 3. In addition, we assumed that mothers usually transfer antibodies to offspring through placenta and breastfeeding (18, 19). Without these antibodies it would be reasonable to assume that newborns would be more prone to infections and hospitalizations than before (20). Thus, in our models, we doubled the number of hospitalized infants (aged 0–12 months) and added them on top of the trend from scenario 3. We chose to double the number of hospitalized infants as a conservative estimate, balancing between a minimal increase and a potentially exaggerated effect.

All forecasts were computed using OLS rather than maximum likelihood estimation as OLS is generally easier to understand and interpret. Further details and assumptions for the scenarios are described in Methi et al., 2022 (5) and its supplementary material.

### 3.2 Evaluation metrics

Forecasted and observed numbers of hospitalizations were compared in terms of peak accuracy and monthly accuracy for the entire period (overall accuracy), distinguishing between cases with and without respiratory support. We applied traditional metrics for evaluating point estimates, such as mean absolute error (MAE) and mean absolute percentage error (MAPE) (24). MAE computes the average of the absolute differences between observed and forecasted points, while MAPE computes the average of percentage differences. Although MAPE offers a more informative comparison when forecasts diverge significantly, we also incorporated MAE as there may be good arguments for penalizing a forecasting of e.g., 200 hospitalizations when 400 are observed harder than 2 hospitalizations when 4 are observed (25).

**Figure 1:**
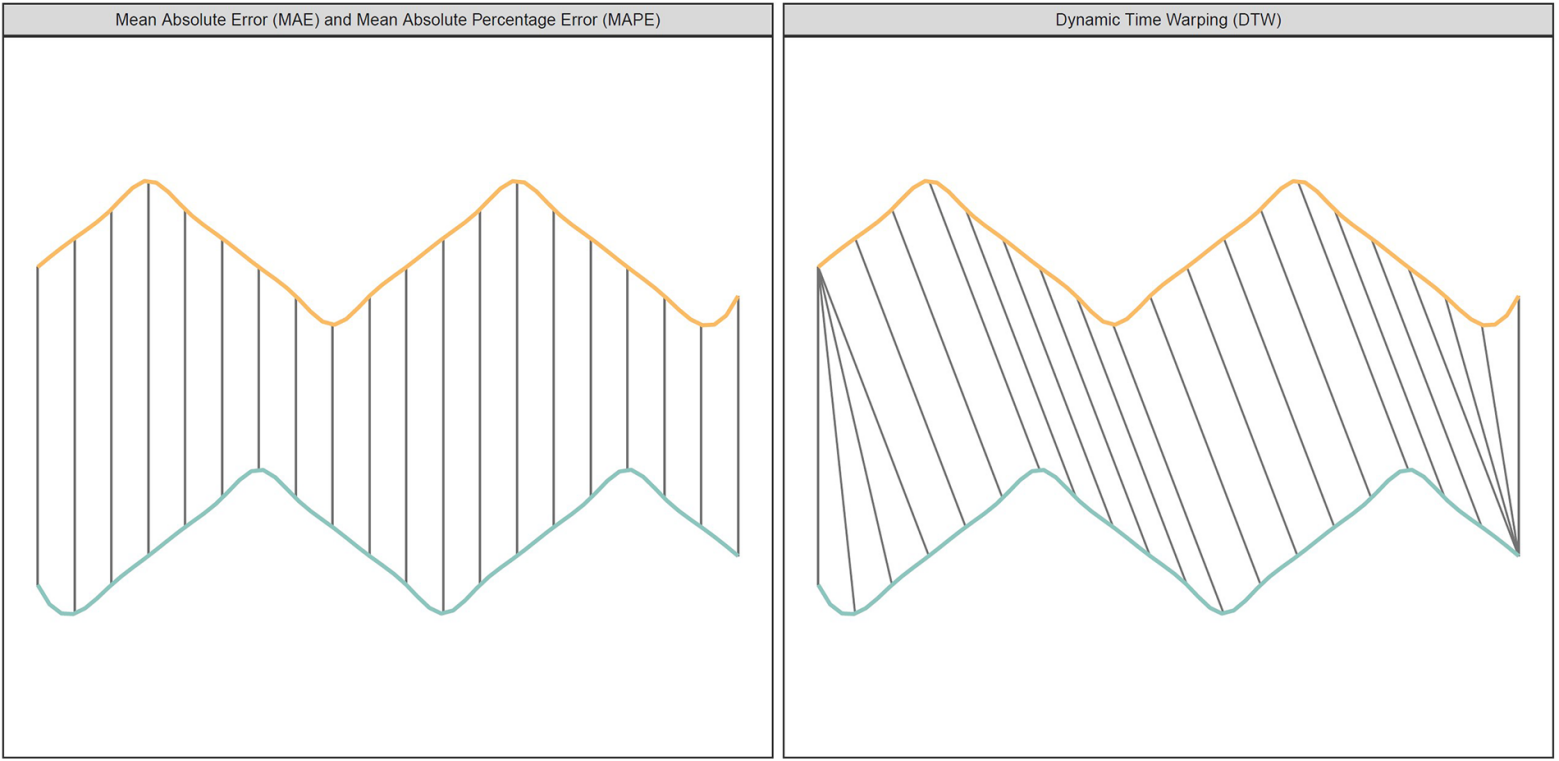
The figure shows two time series (yellow/orange and blue/green) with different peaks. The left panel (Euclidean Matching) shows how traditional metrics compare points. The right panel (Dynamic Time Warping Matching) shows how DTW compare points.

However, neither MAE nor MAPE are suitable to evaluate time series data when there are shifts in trends, as they are both based on comparing the straight-line distance between two points (Figure 1). To overcome this limitation, we utilized the dynamic time warping (DTW) method. DTW is a method that is used to measure the similarity between two sequences that may vary in time or speed. Initially used for speech recognition (26), it has in recent years been more and more applied to other fields such as economics, biology, and database analysis (27, 28). The DTW finds the best alignment between the two sequences by warping them in time. It does this by stretching or compressing the time axes of the sequences to minimize the differences between corresponding points, as shown in the right panel of Figure 1. Once the sequences are aligned, DTW calculates a distance measure that quantifies the similarity. When evaluating the forecast models we applied the Sakoe-Chiba band to avoid slope constraints, which means that our DTW did not restrict the alignment path's slope. While slope constrained DTW only allows a point to connect to the previous, current, or next point, our DTW allowed a point to connect to any other point with the shortest Euclidean distance.

All data handling was carried out in STATA SE v16, and evaluation metrics were computed in R version 4.3.0 using the Yardstick package for MAE and MAPE, and the dtw package for DTW.

### 3.3 Role of the funding source

The study was internally funded by the Norwegian Institute of Public Health by affiliation. No external funding was received. The funding sources had no influence on the study design, the collection or interpretation of the data, the preparation or approval of the manuscript, or the decision to submit the manuscript for publication.

## 4 Results

In total, we included 753,070 children aged 0–5 years between January 2017 and May 2023. On average, we studied the number of hospital admissions among 345,409 children per month. The denominator ranged from a maximum of 363,133 children in February 2017 to a minimum of 329,966 in May 2023. The mean (SD) age for all observations (children-months) was 3.1 (1.7) years and the sample comprised 51% boys.

### 4.1 2017–2023 observed hospital admissions

**Figure 2:**
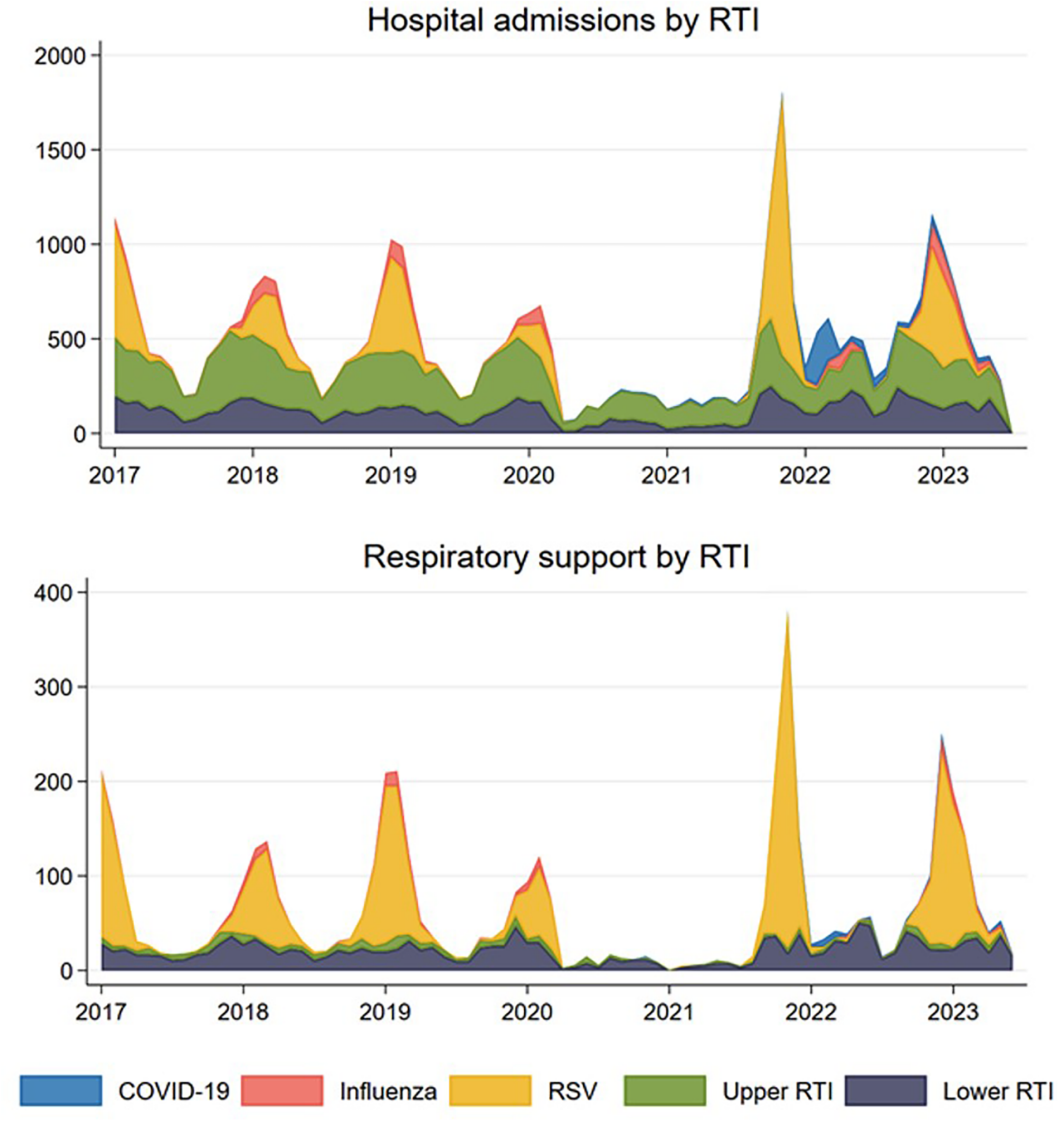
Observed number of monthly respiratory tract infections (RTI) resulting in hospital admission (upper panel) and in need of respiratory support (lower panel) for children aged 0–5 years for the five mutually exclusive groups COVID-19 coded RTI, influenza coded RTI, respiratory syncytial virus (RSV) coded RTI, and other upper and lower RTIs in Norway, January 1st, 2017–May 31st, 2023.

Hospitalization trends due to RTIs showed a consistent pattern from 2017 until the onset of the pandemic in early 2020, with each season being characterized by a monthly peak in January (see Figure 2). Between early 2020 and the fall of 2021, there was a notable decrease in hospital admissions compared to pre-pandemic levels. This was followed by a sharp increase in late 2021 (Figure 2). From Figure 2 it is evident that the prominent peak in late 2021 was primarily driven by RSV. In contrast, the hospitalizations for other types of RTIs (influenza, upper and lower RTIs) remained relatively stable and consistent with their usual levels. It is also evident that the period with the highest number of COVID-19 hospitalizations among young children was at the beginning of 2022.

A similar trend was observed for hospitalizations requiring respiratory support (Figure 2). No peaks were observed during the lockdown phases between early 2020 and late 2021. However, there was an exceptionally high peak during the 2021/2022 season and a slightly elevated peak during the 2022/2023 season. Similar to what was observed for hospitalizations not requiring respiratory support, much of this increase can be attributed to the spread of RSV, rather than COVID-19 or other RTIs (Figure 2).

A noteworthy observation is the deviation in timing of the peak during the 2021/2022 season, manifesting a few months earlier than the normal peak. Instead of the typical peak in January, the highest number of hospitalizations occurred in November. Additionally, the off-season period during the summer was also marked by a higher proportion of hospitalizations than usual. In contrast, the 2022/2023 season displayed a trend more closely aligned with the typical patterns and expectations, as illustrated in Figure 2.

### 4.2 Peak performance of projections

**Figure 3:**
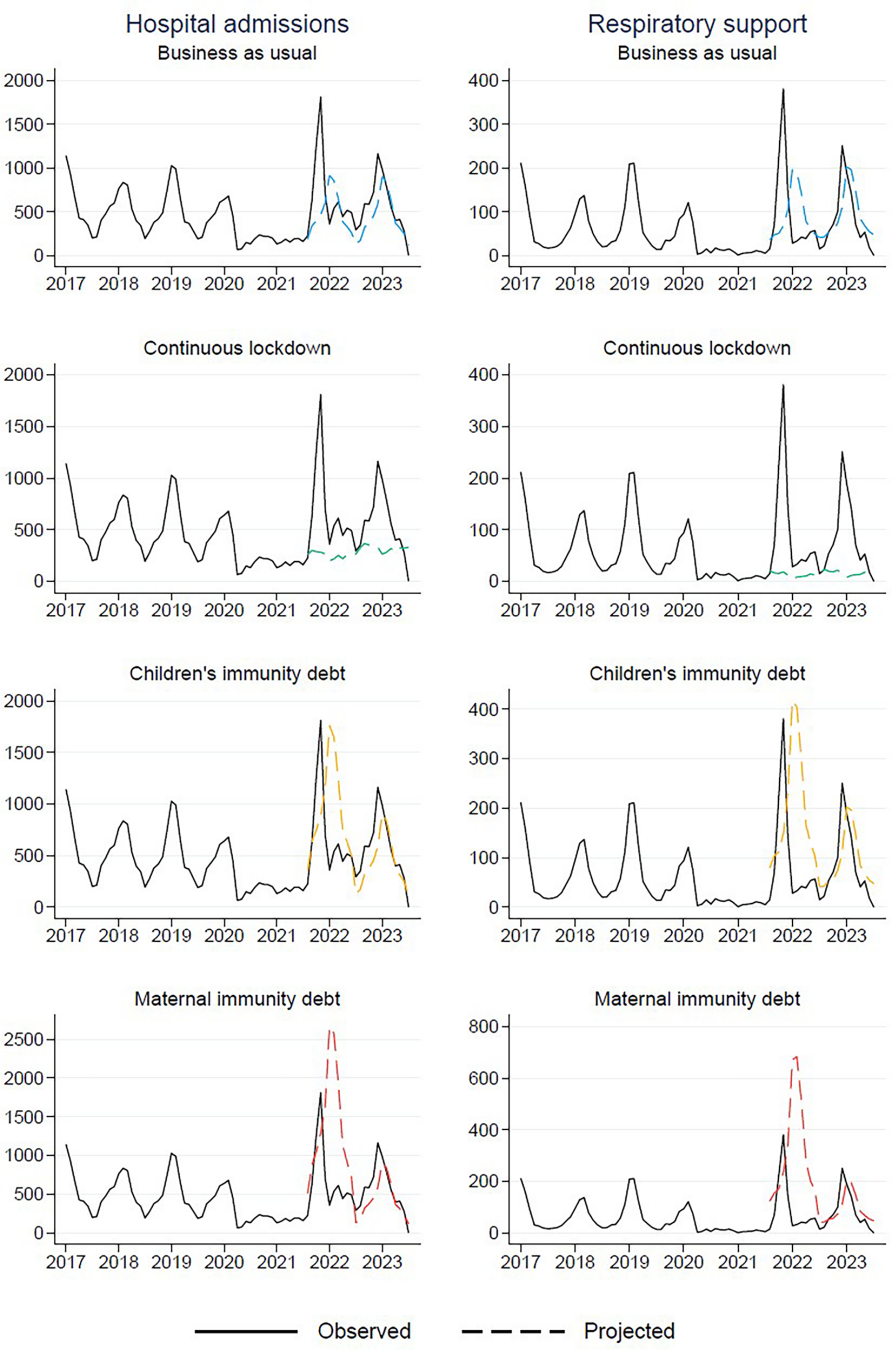
Observed (January 1st, 2017–May 31st, 2023) and projected (from August 1st, 2021 and onwards) number of respiratory tract infections resulting in hospital admission for children aged 0–5 years in Norway.

In the “Business as usual” scenario, where we anticipated a return to pre-pandemic trends, we largely underestimated the overall count of hospitalizations, both with and without respiratory assistance (Figure 3). According to this projection, the peak for the 2021/2022 period was estimated to be 915 hospitalizations, with 197 cases requiring respiratory support (Table 2). However, as the actual peak contained 1,810 hospitalizations and 381 cases requiring respiratory support, this projection fell short by 895 hospitalizations (representing a 49.4% underestimation) and 184 cases requiring respiratory support (48.3% underestimation) compared to the observed data (Table 3).

**Table 2:** Observed vs. projected hospital admissions with and without respiratory support.

Month	Hospital admissions					Respiratory support				
	Observed	Projected				Observed	Projected			
		BAU	CL	CID	MID		BAU	CL	CID	MID
Aug-21	224	185	258	350	509	15	35	23	78	122
Sep-21	631	338	301	640	883	70	47	18	104	154
Oct-21	1,273	393	286	744	1,045	225	50	17	110	169
Nov-21	1,810	466	283	883	1,288	381	67	20	149	235
Dec-21	705	603	264	1,143	1,630	142	104	12	229	341
Jan-22	359	915	197	1,763	2,664	28	197	6	419	686
Feb-22	538	856	216	1,651	2,585	33	190	10	405	675
Mar-22	613	649	252	1,249	1,940	42	141	13	300	511
Apr-22	444	385	218	740	1,133	39	78	9	166	277
May-22	516	330	257	633	914	54	62	14	132	204
Jun-22	492	261	258	501	719	57	48	13	103	162
Jul-22	294	132	264	132	132	15	41	14	41	41
Aug-22	349	169	324	169	169	22	42	28	42	42
Sep-22	592	322	367	322	322	54	53	23	53	53
Oct-22	585	377	352	377	377	71	56	22	56	56
Nov-22	720	451	349	451	451	100	74	25	74	74
Dec-22	1,163	588	330	588	588	251	110	17	110	110
Jan-23	988	900	363	900	900	189	203	11	203	203
Feb-23	783	842	282	842	842	142	196	15	196	196
Mar-23	558	634	318	634	634	70	147	18	147	147
Apr-23	400	369	284	369	369	41	84	14	84	84
May-23	412	313	323	313	313	53	68	19	68	68

Note: The table shows the number of observed and projected 0–5-year-olds in Norway for RTIs per calendar month. The left columns show the total number of hospital admissions. The right columns show the total number of hospital admissions in need of respiratory support. BAU: Scenario “Business as usual”, CL: Scenario “continuous lockdown”, CID: Scenario “Children's immunity debt”, MID: Scenario “Maternal immunity debt”.

**Table 3:** Performance of projections.

Projection	Peak		Evaluation metrics		
	*N*	%	MAE	MAPE	DTW
Hospital admissions					
(1) Business as usual	−895	49,4%	276	39.7	4,960
(2) Continuous lockdown	−1,443	79,7%	371	47.4	8,025
(3) Children's immunity debt	−47	2,6%	346	61.0	4,026
(4) Maternal immunity debt	854	47,2%	510	100	6,848
Respiratory support					
(1) Business as usual	−184	48,3%	68	112	872
(2) Continuous lockdown	−353	92,7%	80	71	1,745
(3) Children's immunity debt	38	10,0%	102	227	850
(4) Maternal immunity debt	305	80,1%	149	372	1,747

Note: The table shows the accuracy of each projected scenario. The Peak columns show the difference between the observed peak and the projected peak in number of patients (*N*) and in percentage (%). Evaluation metrics shows how much the projections missed from August 2021 to May 2023. MAE shows the mean absolute error (how much it missed per month on average in raw numbers), MAPE shows the mean absolute percentage error (how much it missed per month on average in percentage), and DTW shows the optimal (least cumulative distance) alignment between the observed and projected estimates. Lower values mean more precise estimates.

In a similar vein, in the “Continuous lockdown” projection, where we assumed that the reduced levels of RTIs would persist, we also significantly underestimated the actual peaks. This scenario predicted a mere 367 hospitalizations and 28 cases requiring respiratory support at the peak of the RTI season (Table 2). However, these projections fell short by 1,443 hospitalizations (79.7% underestimation) and 353 cases requiring respiratory support (92.7% underestimation) (Table 3).

In contrast, the projection “Children's immunity debt”, where we hypothesized that the proportion of children who remained uninfected and non-hospitalized during the lockdown would be prone to hospitalization in the subsequent season, thus resulting in increased numbers, was more accurate. According to this scenario, the projected peak was 1,763 hospitalizations, with 419 cases requiring respiratory support (Table 2). These estimates aligned closely with the observed peak of 1,810 hospitalizations and 381 cases requiring respiratory support (Table 2). The projection slightly underestimated the total hospitalizations by 47 cases (2.6%) and slightly overestimated the cases requiring respiratory support by 38 (10.0%). In general, it did far better than the other projections (Table 3).

Finally, in the “Maternal immunity debt” projection, which was based on the hypothesis that pregnant mothers who did not transfer antibodies to their newborns would contribute to a doubling of hospitalizations among children aged 0–12 months. However, this projection significantly overestimated the actual peaks. According to this scenario, it was estimated that 2,664 children would be hospitalized, with 686 requiring respiratory support (Table 2). This resulted in an overestimation of 854 hospitalizations (47.2% overestimation) and 305 cases requiring respiratory support (80.1% overestimation) (Table 3).

### 4.3 Overall performance of projections

While the “Children's immunity debt”-scenario outperformed the other scenarios in projecting the peak number of hospital admissions during the season, it fell behind in the overall projection, as evidenced by the performance measures of MAE and MAPE (Table 3). Both the “Business as usual” and “Continuous lockdown” scenarios performed better than the “Children's immunity debt” in terms of overall projection accuracy (Table 3). Again the “Maternal immunity debt” largely overestimated the actual numbers (Table 3).

While the three first scenarios performed rather similarly, none of them did well on the MAE and MAPE metrics, with mean absolute percentage errors varying between 39.7% and 61.0% on hospital admissions, and as much as 71% to 227% on hospital admissions with respiratory support (Table 3).

**Figure 4:**
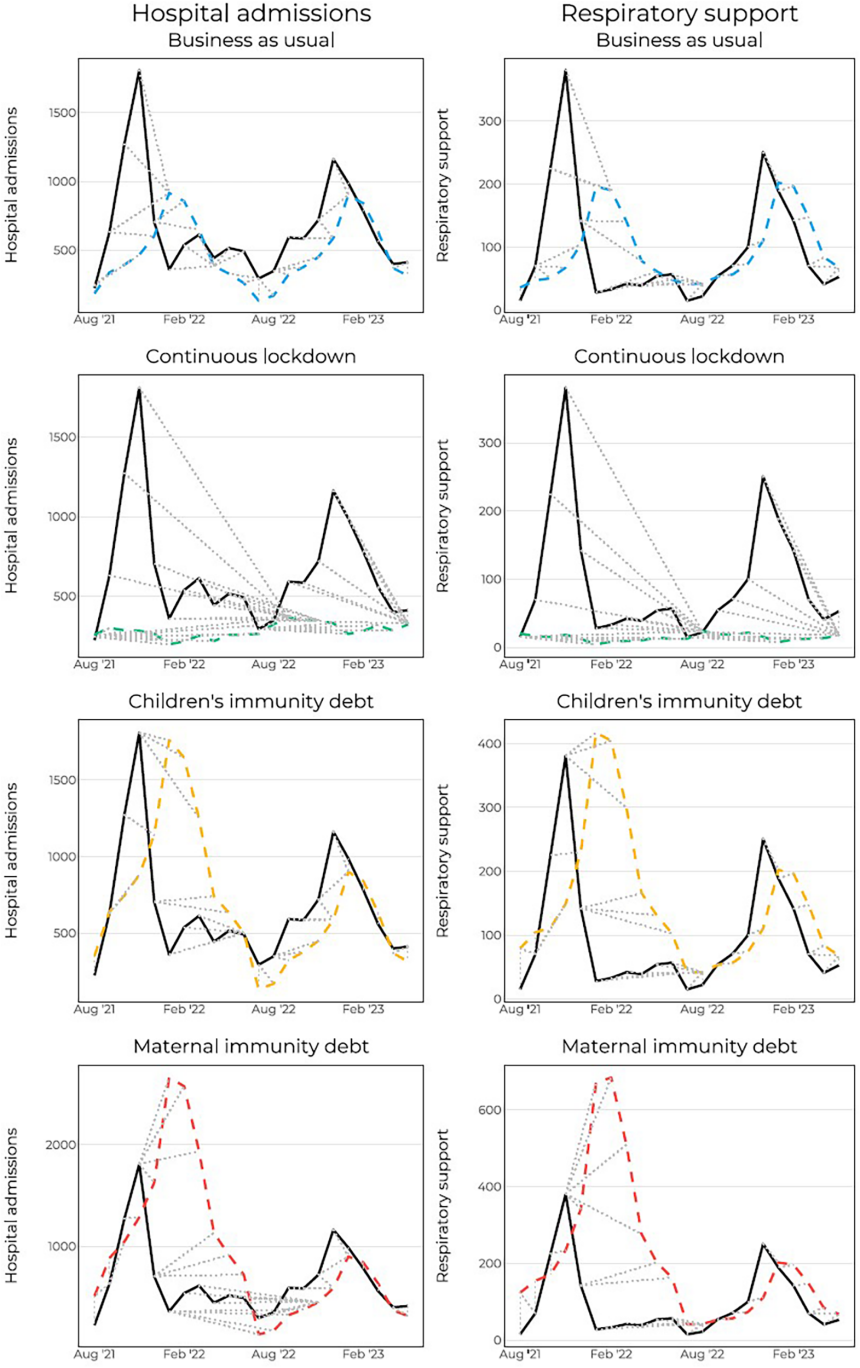
Dynamic time warping for hospital admissions and hospital admissions with respiratory support. Dashed grey lines show which points are connected to which point.

To account for the shifts of the curve, we applied the DTW to estimate the shape of the curve rather than the exact timing of the peaks. When measuring the DTW-distance, “Children's immunity debt” again outperforms the other scenarios (Table 3). This lends the fact that the shape of the curve fits better with the actual data, though the exact timing of the forecasted points was off. Figure 4 shows how each forecasted point (dashed colored lines) are compared with the observed points (solid line) after the warping in DTW was conducted. The dotted grey lines connect the points.

## 5 Discussion

### 5.1 Main findings

In this study, we have evaluated four distinct forecast models designed to predict the subsequent RTI season following the COVID-19 pandemic. Each model contained unique assumptions about how the NPIs during the pandemic would affect the upcoming season. Among our models, the “Children's immunity debt” scenario demonstrated the highest accuracy in estimating the peak monthly number of hospital admissions. This scenario operated on the assumption that the same number of children aged 0–5 years, who did not require hospitalization during the pandemic compared to a typical RTI season, would be hospitalized in the subsequent season. This scenario also offered the most precise depiction of seasonal dynamics, particularly in terms of the shape of hospitalization curves, as measured by DTW.

Since all forecast models were based on dynamics from previous seasons, they all projected the peak to occur in January. However, the peak of the 2021–2022 season was observed in November. When assessing the raw point differences month for month using MAE and MAPE, both the “Business as usual” and “Continuous lockdown” scenarios performed comparably to, or even better than, the “Children's immunity debt” scenario.

We also observed that the highest share of hospitalized children for COVID-19 occurred at the beginning of 2022. This may be due to the emergence of the Omicron variant, which exhibits a higher propensity for infecting children when compared to other strains of the virus (3), and at a time when most of the adult population was vaccinated with at least two doses, and older children had started receiving the vaccine (29).

### 5.2 Previous studies

To the best of our knowledge, this is the first study to investigate the performance of different forecast models for hospitalizations, encompassing various RTIs that typically affect young children. Previous studies have predominantly focused on separate pathogens, such as influenza forecasting (30), RSV forecasting (31), or COVID-19 forecasting (32), not including several RTIs in the same models. This is important as health authorities and hospital planners may be more concerned with hospital capacity management, avoiding overload, and optimizing the allocation of public health resources, rather than with the precise behavior of specific pathogens. When comparable studies exist, they generally contain a single model (33), often over brief time frames (13), and without testing different assumptions. In addition, existing forecasts are often complex and grounded in data that may be challenging for policymakers and non-statisticians to grasp. What our study offers is a straightforward forecasting approach, based on simple assumptions: returning to pre-pandemic levels (business as usual), maintaining suppressed RTIs (continuous lockdown), considering immunity deficits among children (children's immunity debt), and accounting for amplified deficits among infants (maternal immunity debt).

While traditional point estimates have often been evaluated using metrics such as MAE, MAPE, the root-mean-square error (RMSE), or correlation coefficient, other disciplines have looked at DTW for evaluating cases when timing differs (27, 28). To our knowledge, this is the first paper to evaluate epidemiologic forecasts using DTW. We demonstrate that using DTW can be beneficial for evaluating forecast models where the observed values are similar in shape to the forecasted values but differ in timing. Depending on researchers’ objectives, we argue that using DTW is valuable for evaluating the overall seasonal dynamics and shape. However, if the focus is on predictive accuracy, such as pinpointing the timing of peaks, DTW may not be the most suitable metric.

Besides our methodological contribution, one of the most important findings of our study is the increase in hospitalizations among children after the lockdown measures were eased. This is often termed “immunity debt”. The notion of an immunity debt has garnered discussion and remains a topic of ongoing debate among immunologists and infectious disease experts (34). While our findings support the existence of an immunity debt among children, further research is needed to gain a comprehensive understanding of the effects of pandemic-related measures on the development of children's immune systems. Future studies should explore the potential long-term consequences and implications of immunity debts.

Our findings of an increased number of RTI hospitalization following the ease of restrictions are also consistent with data obtained from several other countries, such as New Zealand and Australia (16, 35), Japan (36), Denmark (37), and Sweden (38). Together, these data support the presence of an epidemiological rebound. Furthermore, although not a primary aim of our paper, our findings underscore the low burden of COVID-19 placed on hospitals by young children compared to other common causes of RTIs, such as RSV and influenza (Figure 2).

### 5.3 Interpretation and relevance

The primary policy relevance of this study is to highlight important assumptions that can serve as a planning tool for healthcare services during crises, when public health resources are scarce. The straightforward calculation of forecast models proposed in this study enhances the accessibility of the research to a wider audience, including policymakers and healthcare professionals. By clearly outlining the methodology and assumptions used in projecting the RTI admissions, the study ensures transparency and reproducibility. This transparency enables other researchers to validate and replicate the calculations, further strengthening the reliability of the study's findings.

Although we conclude that the scenario of an immunity debt best projected the upcoming season after lockdown measures were eased, other assumptions may be more accurate under different circumstances. However, the insight gained from this study makes both researchers and policy makers better prepared for future pandemics when NPIs are introduced or are planning to be introduced.

### 5.4 Strengths and limitations

A major strength of this study is the utilization of register data, which allows for the inclusion of the entire population of 0- to 5-year-old children in Norway. This approach helps minimize selection bias, as it encompasses a broad and representative sample of children aged 0–5 years in the country. By including the entire population, the study achieves greater internal and external validity, enhancing confidence in the study's findings and reducing the potential for sampling errors. The use of register data also provides a comprehensive and reliable source of information, as it is collected systematically and consistently. This enhances the accuracy and completeness of the data, allowing for a more robust analysis of RTI hospitalizations and improving the overall quality of the study. An additional strength is that our findings may be applicable to countries with similar healthcare systems, such as Norway.

This study also has limitations. First, we only included new hospital admissions, and we did not account for the length of hospital stays for RTI admissions. The duration of hospitalizations is an important factor that could influence the overall burden of RTIs, and the healthcare resources required. Therefore, this study's findings should be interpreted in conjunction with other factors that may impact the length of hospital stays. While we have considered respiratory support as an indicator for the cases with the highest healthcare needs, it is important to recognize that the needs of RTIs can vary across different hospital admissions. The inclusion of respiratory support provides insight into severe cases, but it may not capture the full spectrum of severity and the potential variations in outcomes.

Another limitation in our study is the potential impact of co-infections and interactions between different RTIs. Co-infections can complicate both diagnosis and interpretation, as multiple pathogens may simultaneously contribute to the clinical presentation and severity of an RTI. In this paper, we addressed co-infections by including only the most specific pathogen identified in our dataset for each case. For instance, if a patient was diagnosed with both a specific virus, such as RSV, and an unspecified lower RTI, the case was categorized as an RSV-coded RTI. While this approach simplified our analysis and ensured that each case was counted only once, it may have undermined the broader complexity of co-infections and interactions.

A second limitation is that although our findings support the notion of an immunity debt, we cannot counterfactually state that the children hospitalized would have been hospitalized in the previous year had it not been for the non-pharmaceutical interventions and lockdowns. Similarly, variations in healthcare access and changes in individual behaviors could contribute to fluctuations in RTI hospitalizations and may have influenced the observed patterns. For the latter, this should only affect the results to a minimal extent given that the study focuses on “hard” measures such as hospital admissions and in particular respiratory support.

## 6 Conclusions

In scenarios similar to the COVID-19 pandemic, when RTIs are suppressed for a longer period, a simple regression model assuming that the same number of non-hospitalized in a normal season would be hospitalized in the following season, forecasted the hospital admission numbers most accurately. This simple forecast may be useful for capacity planning activities in hospitals.

## Data Availability

The datasets presented in this article are not readily available because the individual-level data used in this study is not publicly available due to privacy laws. However, the individual-level data can be applied for through helsedata.no/en. Requests to access the datasets should be directed to https://helsedata.no/en.
